# Training, Wellbeing and Recovery Load Monitoring in Female Youth Athletes

**DOI:** 10.3390/ijerph191811463

**Published:** 2022-09-12

**Authors:** Dani A. Temm, Regan J. Standing, Russ Best

**Affiliations:** Centre for Sport Science and Human Performance, Waikato Institute of Technology, Hamilton 3200, New Zealand

**Keywords:** injury, overreaching, menstrual cycle, GPS, RPE, LTAD

## Abstract

Participation in youth sports is ever-increasing, along with training and competition demands placed upon youth athletes. Young athletes may experience high training loads due to playing several sports, as well as participating in school physical education. Therefore, monitoring youth athlete load is an emerging area of research that may help limit non-functional overreaching, injury, or illness and assist with long-term athlete development. This narrative review highlights that multiple measures have been explored to monitor both internal and external load. However, the validity, reliability and practicality of these measures are often not fully understood in female youth populations. The most commonly used external monitoring methods are GPS tracking and TRIMP whereas common internal monitoring tools are questionnaires, perceived exertion rating and heart rate measures. The reporting of injuries and menstrual cycles is also crucial for providing completeness when monitoring an athlete. It has been suggested that the combination of training load, recovery and wellbeing monitoring variables is the optimal way to monitor an athlete’s fatigue levels. Whichever monitoring method is applied, in a youth population it is important that the protocol can be individualised, is inexpensive and can be easily implemented and reported so that the monitoring is sustainable.

## 1. Introduction

Athletes are subject to a plethora of external and internal stressors and it is the relationship between these stressors such as training load, psychological well-being, and recovery that can not only affect their performance but are also critical for the mitigation of injury and illness [1]. This relationship becomes increasingly more intricate in youth athletes as a result of additional stressors such as multiple sporting obligations, schooling, social interactions, and physical maturation [2]. Given the different stressors that arise with youth, it can be suggested that there are different intentions to monitoring in comparison to elite athletes. Both contexts intend to preserve athlete wellbeing and ensure that optimal recovery is being facilitated to prevent injury. For a youth athlete, monitoring should also assist long-term athlete development [3,4]. The International Olympic Committee consensus statement on youth athletic development has set a clear goal to “develop healthy, capable and resilient young athletes while attaining widespread inclusive, sustainable and enjoyable participation and success for all levels of individual athletic achievement” [5]. However, the reality is, with an increase in both training and competition, these athletes are partaking in sizable and rigorous training volumes as a result of playing several sports [1].

Many youth athletes are not only committed to one sport and/or team. They may be competing in a school team and a club or representative team in one or more sports, whilst also regularly exercising through school physical education [6]. Nonetheless, it is through monitoring that healthy athletes can be developed, as information is available to assist coaching staff in deciding whether an athlete is physiologically, biomechanically, and psychologically ready to train or compete to the best of their ability [1]. In doing this, youth athletes are less likely to become injured or experience burnout, enabling them to be involved in sports for a longer duration [7,8]. A multi-year study prospectively following 372 students from a European school, accounting for 16 different disciplines, reported that approximately 20% of injuries resulted in an absence from sport for four or more weeks, with nearly 40% of injuries occurring as a result of chronic overuse [9,10]. These chronic overuse injuries are on the rise as more children are participating in competitive sports from a younger age [11]. *Advances in Paediatrics Journal* has outlined common paediatric overuse injuries such as Little League shoulder, Little League elbow and gymnast wrist, which begins to showcase the increasing prevalence of overuse injuries in these sports [11].

As well as high injury rates in youths, burnout rates are also increasing. Burnout is defined as an athlete ceasing to participate in a previously enjoyable sport as a result of chronic stress [7]. Gustafsson et al. [12] investigated the prevalence of burnout in youth athletes using the Eades Burnout Inventory. Across both female and male athletes participating in 29 different sports at a school sports level, it was found that most young athletes admitted to experiencing low to moderate levels of burnout, with prevalence increasing as age increases [12].

Injury, burnout and performance are all influenced by fatigue. Fatigue is often described as being multifactorial, as it has many different possible mechanisms and effects, resulting in various definitions being used [3]. A prevailing definition is failure to maintain the needed force or to have a diminished capacity for maximal performance [3]. However, a more practical definition is the inability to fulfil a recently attainable task [13]. As fatigue is multifactorial, variables such as stress, training load, nutrition, hydration and sleep quality considerably influence fatigue [14,15]. Consequently, monitoring of these variables and the subsequent fatigue needs to encompass performance, biomechanical, physiological and subjective measures as all may be adversely affected by training load. Yet this is a two-way relationship as fatigue also influences the desired training load as prior fatigue can inhibit an athlete from reaching set out targets within their training, showcasing how training load monitoring needs to be considered both retrospectively and prospectively.

Training load monitoring observes and analyses internal and external load using such tools as rating of perceived exertion, questionnaires, countermovement jumps, Global Positioning Systems (GPS) tracking, and/or repeated sprints [1,3,16,17,18]. At present, there is a disparity in studies published which investigate training load monitoring and other correlating factors such as overuse injuries or the prevalence of burnout in female and male athletes [19]. As a result, there are limited discussions regarding how males and females may require different monitoring needs, causing practitioners to apply evidence developed on male athletes to females [20]. This disparity is further amplified when discussing the monitoring of female youth athletes. Monitoring female youth athletes not only encompasses the complexities seen in youth monitoring of possible multiple sporting commitments, schooling and other social, emotional and psychological developments; there is also the added complexity of the menstrual cycle and its effects on training [2]. This may add new physical and mental stress leading to changes in training and recovery abilities [21]. Therefore, this narrative review will discuss current monitoring practices for athletes, focusing on youth female athletes as the literature allows. The practicality of monitoring these variables in (female) youth athletes and their validity and reliability are also considered, plus areas for further research. At the end of the review, we provide a summary figure collating the findings from each monitoring factor. We also depict key considerations for each factor, as well as gold standard and green standard monitoring practices for each factor. Gold standards are considered the most reliable and valid, but may incur additional cost that may be unrealistic in some youth performance settings; hence, a green standard option is a low cost alternative. In some cases, gold and green standard practices overlap.

## 2. Literature Review

### 2.1. Youth Athletes and the Long-Term Development Model

Youth participation in sports and other physical activities varies depending on such things as country, age, gender and socioeconomic background. In Australia, it was reported that 60% of 5- to 14-year-olds and 28% of 15-year-olds and older participated in at least one organised sport outside of school [22], whereas in American it was reported that approximately 50 to 56% of 6–12-year-olds and 54% of high school student participated in a sport at least once a year [23]. What is commonly seen, however, is that participation in sports and physical activity typically peaks between 10 and 14 years of age before declining between 15 and 19 years of age [24,25]. Youth athletes are characterised as either children or adolescents. Children are up to 11 or 13 years old for girls and boys, respectively, with adolescents comprising the remaining ages until 18 years old for both sexes [26].

The drop in participation seen between 15 and 19 years old may be attributed to several aspects such as the change in participation to specialisation, an increase in school workload or other commitments, the removal of compulsory physical activity in school, the decrease in social play or injury and/or burnout [27]. As a result of the increasing number of children and adolescents playing sports, guidelines have been created to advise a way to train youth with the intention of increasing the longevity of youth athletes. The long-term athlete development (LTAD) model aims to optimise training, competition and recovery for the different stages of athlete development, identifying significant phases of accelerated changes to training [28]. Specialisation, where youth athletes transition from playing several sports at a time to pursuing one specific sport, has been explored thoroughly in the realm of LTAD. An individual either specialises early, typically under 12 years old, or specialises late, over 12 years old [28,29,30]. There are sports such as figure skating, diving or gymnastics where early specialisation may be necessary as elite-level competitions begin before full maturation [7,31]. However, through the LTAD framework, it is encouraged for youth athletes to specialise late as this may decrease the rates of overuse injuries and burnout [7,28]. It has also been seen that young female athletes that specialise early may develop coordination and motor deficits in comparison with multisport athletes, which may mean that coaches need to consider programming integrated neuromuscular training to mitigate this risk [32]. Conversely, it has been reported that early specialisation in one sport does not increase the odds of reporting an injury history; however, the exceedance of the recommended sports participation volumes does [33].

The pathway of late specialisation consists of six phases; FUNdamentals, learning to train, training to train, training to compete, training to win, and retirement/retainment [28]. These different phases use the onset of Peak Height Velocity (PHV) as a reference point to program regarding the critical and sensitive stages of trainability during the maturation process [28]. The ages at which individuals reach each stage will differ as a result of the individual’s sex and personal maturation rate. Females typically reach PHV at 12 years old, whereas males at 14 years old; however, some youth mature earlier, often up to a year before the average PHV age [34]. Youth athletes before the average PHV age, typically between the ages of 6 and 14, have been reported to experience limb mass increasing twice as much as limb length [35,36]. This can add to the imbalance between flexibility, strength and force generation of cohesive muscle groups [35,36]. Therefore, for youth athletes, factors such as hormonal, musculoskeletal, neurological and anatomical changes as well as the degree of maturation must be considered when designing training and recovery protocols [28]. Thus, there is not only the need to monitor maturation changes but also the need to monitor other variables such as menstrual cycle, training load, physical stress, recovery and general wellness to ensure athletes are not only achieving performance increases but are managing the risks of injuries and burnout.

### 2.2. Menstrual Cycle Monitoring

**Figure 1:**
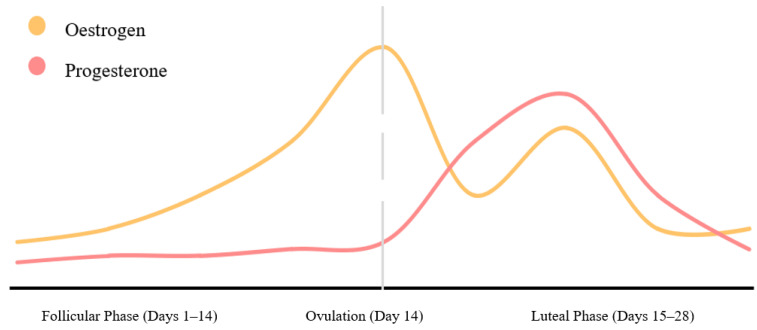
Stages of the menstrual cycle and associated hormonal fluctuations. **Note**: Grey line highlights the halfway point of a standard 28-day cycle. It is at this point that Oestogren is at its peak.

The female menstrual cycle is an often overlooked and underexplored variable in sport, particularly from a monitoring perspective. The menstrual cycle is approximately 28 days long and is divided into two stages, the follicular phase and the luteal phase, with ovulation in between (Figure 1) [37]. During each stage, several physiological changes occur that may reduce or heighten the physical performance of an athlete [38].

The primary hormones involved in the menstrual cycle have been shown to impact substrate metabolism, ventilation, the cardiovascular system, and thermoregulation [21], although current literature surrounding the extent of the effect the menstrual cycle has on athletic performance is inconclusive. Sarwar et al. [39] found that there was a significant increase (11%) in handgrip and quadricep strength during the middle of the athlete’s cycle (roughly days 12 to 18) in comparison to the luteal and follicular phase, whereas a study by Romero-Moraleda et al. [40] found that there was no significant difference in overall force, velocity and power between the menstrual phases. Similarly, an investigation by Paludo et al. [41] highlighted a significant change in aerobic performance throughout the menstrual cycle phases, which opposes the findings of studies such as Sunitha et al. [42].

The fluctuations in associated hormones have been suggested to be a risk factor for the occurrence of non-contact anterior cruciate ligament (ACL) injuries [43]. A 2017 systematic review of 17 studies concluded that there was strong evidence suggesting that females are at a greater risk of an ACL injury during the pre-ovulation phase in their cycle, predominantly due to hormonal effects on joint laxity [44]. These results were supported by a further review, determining that ACL laxity and therefore injury risk increases during ovulation, as well as reporting that oral contraceptive use may reduce injury risk by 20% as a result of limiting hormonal fluctuations [45]. Both reviews stated that there is a lack of high-quality studies in this area and significant methodological differences limit application and consistency of findings.

Menstrual cycles can be used as a health marker in female athletes. The female athlete triad is a spectrum of interconnected conditions and problems such as low energy availability, with or without an eating disorder, low bone mineral density and menstrual dysfunction [46]. Menstrual dysfunction or menstrual irregularity includes primary amenorrhea, the delay in menarche typically after the age of 15, secondary amenorrhea, the cessation of menstruation for 3 or more consecutive months after regular menses, and oligomenorrhea, a cycle greater than 35 days apart [47,48,49]. A 2011 study investigated the prevalence of menstrual dysfunction in 311 female high school athletes found that 18.8% reported a form of menstrual irregularity in the past 12 months [49]. Similar results were seen in a study by Nichols et al. [48], wherein a cohort of 170 female high school athletes, the prevalence of menstrual dysfunctions was 23.5%.

Menstrual dysfunction and its effects on bone mineral density is problematic in youth as 25% of bone mass accumulates during the two years surrounding the first menstrual cycle, with roughly 90% of peak bone mass reached by the age of 18 years old [46]. Research has shown those female athletes who have had amenorrhea for greater than one year as an adolescent were 23 times more likely to develop low bone mineral density as an adult [50]. Along with decreased bone mineral density comes an increased injury risk which was illustrated in a study of 136 female youth athletes as those with low bone mineral density were over three times more likely to incur an injury [51]. In the same cohort, it was determined that athletes with oligomenorrhea or amenorrhea exhibited an almost three-fold increase in injury risk than those with a regular menstrual cycle [51]. The combination of both the menstrual cycle’s effect on performance as well as the risk of menstrual dysfunction illustrates not only the need for screening of menstrual cycle irregularities but also monitoring to optimise training and recovery for the individual athlete.

Menstrual cycles can be recorded/monitored through many methods such as a diary, questionnaires, app recording, or basal body temperature recording. Calendar-based counting, which is used in a diary or virtual application, is a method of reporting and establishing phases of the cycle through self-reporting the onset of menses as day one and counting days from that point to determine such things as ovulation [52]. This provides limitations as the length of the follicular phase is more prone to variation than the luteal phase, meaning that unless the cycle is retrospectively observed, it can be difficult to accurately predict when the phases will occur [52]. This method also assumes that the individual has a regular menstrual cycle, and it is common for menstrual cycles to be irregular or over 35 days in length in the first five years after beginning menstruation [49]. Basal body temperature (BBT) charting is another widely used method to determine phases within the menstrual cycle. This method requires an individual to measure their body temperature with a thermometer that is sensitive enough to measure 0.05 °C change, first thing in the morning [52]. Throughout their cycle, fluctuations would occur in BBT as after ovulation many women experience an increase in BBT of roughly 0.3 °C throughout the luteal phase; however, some women do not experience this fluctuation and readings can be influenced by factors such as stress, medications, illness, and sleep [53,54]. The most direct ways to monitor ovulation and therefore different phases of a cycle are serial follicular scanning or hormone analyses through blood, serum, urine or saliva; however, these methods are often time-consuming, expensive and invasive [52]. For example, salivary hormone analysis allows monitoring of hormones throughout the menstrual cycle [21,55]. One study used this method to determine menstrual cycle phases when exploring their effects on V0_2_ max and associated cardiorespiratory dynamics [56]. This method of testing and monitoring is non-invasive and convenient as it is able to be self-collected as well as stored in a home freezer before being delivered to a laboratory for analysis [52,55]. Due to this convenience, this method is often performed daily to achieve precise identification of each phase and subphase [57]. Initial studies using salivary progesterone analysis suggested it was a poor marker of ovulation [58]. However, subsequent studies have shown salivary progesterone and oestrogen levels are an appropriate method to determine menstrual phases [59]. Regardless, limitations do still exist with these types of methods, especially within a youth population. The availability of getting the saliva samples analysed through a laboratory is the primary limitation due to cost and accessibility to laboratory facilities for youth sports as funding is often scarce. Secondly, there is high variability in hormone levels between individuals, especially in a youth population where their menstrual cycle is often not yet regular [57]. This, therefore, hinders the establishment of average reference ranges to determine each phase. Overall, salivary hormone analysis provides a convenient and acceptable method of identifying menstrual phases. However, due to the resources typically available at youth level, this method is unlikely to be applicable in this setting. Therefore, when the aim is to monitor and report menstrual cycles of female youths, a method such as counting back may be best suited to this population as it is quick, easy, non-invasive and does not cost.

A consideration that needs to be acknowledged when exploring menstrual cycle monitoring is the lack of knowledge in youth athletes surrounding what is a regular menstrual cycle and aspects such as what defines the beginning of a new cycle. This issue can lead to athletes inaccurately reporting, potentially leading to the missed or wrong identification of menstrual dysfunction linked to relative energy deficiency in sport (RED-S) [60]. This can be a result of irregularities in menstrual cycles in youth caused by a cycle that is not fully developed or as a result of energy deficiency as seen in RED-S [61]. A final consideration when monitoring menstrual cycles in a youth population is that these athletes may not be comfortable discussing menstrual cycles and therefore do not report any changes in their cycle. Brown et al. [62] explored elite female athletes’ experiences and perceptions of the menstrual cycle regarding sport and training. It was reported by participants in this study that they felt awkward and uncomfortable discussing menstruation with coaches, in particular a male coach [62]. Subsequently, participants reported that if they gained more knowledge and understanding surrounding the effects of the menstrual cycle on their performance, they may be more comfortable talking to their coach about it [62].

### 2.3. Wellbeing Monitoring

Athlete wellbeing explores the wider aspects of an individual’s life, such as stress, sleep, hunger, work or school and their impact on an athlete’s total load, thereby influencing their ability to perform and recover optimally [63]. Most commonly, questionnaires are used to assess an athlete’s perception of these variables as they are effective, simple and inexpensive systems to evaluate an athlete’s load. Malone et al. [64] observed the impact of elite soccer players’ perceived well-being on their training output and concluded that there were significant effects of pre-task wellbeing on external and internal training load output measures, suggesting that players’ perceived well-being before training can provide important information regarding their expected training output.

In youth athletes, wellbeing monitoring in addition to training load monitoring is particularly important, as youth athletes commonly face stressors from other sources such as school, relationships and pressure from both coaches and parents [65]. Youth athletes’ stress is often cyclical, as an athlete’s perceived stress level increases gradually throughout the year as academic requirements increase, culminating during exam period(s), after which it significantly decreases [63]. Excessive stress, either training or non-training related, can increase the risk of injury and illness, putting athletes at risk of overtraining and burnout, ultimately affecting an athlete’s health [66,67,68].

Illness accounts for ~14% of lost training and playing days, which are notably higher during times of excessive stress such as exam periods [63]. A significant downfall of some wellness questionnaires is the time-consuming length and lack of specificity when using a pre-set questionnaire [69,70]. For this reason, the most frequently adopted tool to assess wellness are customised questionnaires that allow flexibility in the questions asked and scales used, creating a tool that is specific to the athlete or team’s needs [13,71]. Customised questionnaires are usually adapted from existing questionnaires which were used successfully in prior studies [63]. Customised questionnaires typically comprise four to twelve variables measured on Likert scales ranging from one to five or one to ten [70]. These questionnaires often assess mood, sleep quality, stress levels, muscle soreness and overall fatigue [63,72]. Many teams and sporting codes have adopted their own version of wellness questionnaires into monitoring practices as a method of promoting compliance and increasing specificity [73,74,75,76,77]. Gastin et al. [69] investigated the efficacy of self-reported ratings of wellness using a customised questionnaire in elite football players. The athletes’ self-perceived ratings of wellness were sensitive to both the daily and weekly variations in recovery and wellbeing status [69]. Whilst these customised questionnaires are undoubtedly advantageous from a practical standpoint, it has been argued that the customisation of these questionnaires without appropriate testing can impede their scientific and statistical reliability and validity [71]. Another downfall of all subjective measures is the influence of athletes’ experience with self-monitoring, and the potential ability to manipulate results by over/underestimating their recovery levels due to a lack of awareness of their limits across different bodily systems. There is currently a paucity of female-specific literature that explores the use of these questionnaires and wellbeing monitoring tools within this population. This, therefore, means that recommendations regarding these tools are made on the assumption that females and males’ respond comparably. However, overall, wellness questionnaires can be an efficient and inexpensive monitoring tool to analyse variables outside of training stressors, assuming appropriate reliability and validity is maintained. It is recommended for these to be used in conjunction with objective methods of internal and/or external load to fully understand an athlete’s holistic state.

### 2.4. Training Load

Training load describes the quantification of ‘work’ athletes are performing, whilst mitigating unnecessary fatigue and enhancing performance [13]. Monitoring training load provides coaching staff with a better understanding of whether an athlete is physiologically and biomechanically ready to train or compete at the desired levels [1]. In youth athletes, this is of great importance due to the emphasis on both injury prevention and LTAD. Training load can be split into two categories, either internal or external. Internal load evaluates the relative physiological and psychological stressors inflicted on the athlete [78]. Common measures for internal load are rating of perceived exertion, heart rate or blood lactate values [13]. Measuring external load refers to the objective collection of athletic performance data that is independent of internal measures [3]. These include GPS parameters, power output testing or time-motion analysis [1]. Each method of monitoring training load provides a slightly different perspective on the training loads’ impact on the athlete in their specific sporting context. Within this section of the review, RPE, TRIMP, ACWR and GPS are explored as they are the more prevalent monitoring methods in the literature [3,13].

A commonly used method of assessing internal load is the rating of perceived exertion (RPE), specifically session RPE (sRPE). RPE assesses retrospective load through individual athletes’ subjective ratings of their own perceived exertion during training or competition, typically using the Borg scale of 6–20 [3]. This system has been further adapted to include session duration as a potential solution to the problems associated with measuring heart rate during training and competitions. sRPE uses the same retrospective subjective rating of exertion; however, it is obtained 30 min following the end of the training or game and uses the CR-10 scale [79]. The overall RPE rating is then multiplied by the session duration to give a quantifiable measure of an athlete’s internal training load [72]. Previously, sRPE has been correlated with summated heart rate zones during exercise (r = 0.75–0.90), and heart rate training impulse (r = 0.65–0.91) [80,81]. sRPE has also been shown to be a sensitive marker (*p* ≤0.05) of internal training load during a competitive training cycle and reliable across different training modalities such as strength training (ICC = 0.88) [82,83]. The validity of sRPE has been explored in elite youth football players when analysed alongside the Banister training impulse. Very large correlations were seen at a group level (r = 0.77) and an individual level (r = 0.70–0.95) [84]. Additional data also suggest retrospective measures taken after 24 h maintained a nearly perfect correlation (r = 0.97–0.99) with measures taken 30 min post-training within a youth population [85,86].

RPE is not without limitation, as this global score may not be sensitive enough to thoroughly monitor a range of both physiological and biomechanical exertion signals during exercise [87,88]. Another limitation of sRPE is determining the smallest worthwhile change or the threshold where changes in the outcomes are sufficient to have a significant negative or positive impact on performance and health [3,70]. In using sRPE, additional monitoring tools and/or systems may be needed to determine this threshold. Furthermore, a lack of understanding of true maximal exertion, often seen in youth athletes, can mean their ability to self-assess can be unreliable [1].

Another common method of monitoring internal training load is through the use of heart rate, specifically training impulse (TRIMP). Bannister first developed the TRIMP method, which used duration, fractional elevation in heart rate and a weighting factor to represent changes in exercise to quantify the internal load of a session [18]. This method has been further developed over time to better reflect the heart rate workload during intermittent exercise [89]. Edward’s TRIMP method assesses time spent in five arbitrary heart rate zones multiplied by the correlation coefficient and summed for a quantifiable training load measure [18], whilst Lucia’s TRIMP model uses three heart rate zones that are based on an individual’s tested lactate threshold and the onset of blood lactate accumulation [3]. This version of TRIMP, and other similar individualised TRIMP (TRIMPi) based metrics have been considered to be stronger models of assessing train load due to the tailored approach they provided [90]. A 2004 study on young soccer players reported individual correlations between Foster’s RPE measure of training and various TRIMP methods [91]. Banister’s TRIMP reported a 0.5–0.77 correlation, Edward’s TRIMP reported a 0.54–0.78 correlation and Lucia’s TRIMP reported a 0.61–0.85 correlation, where <0.5 is unreliable, ≥0.8 is reliable ≥0.9 is very reliable [92].

TRIMP may also be a valuable monitoring tool in sports where GPS cannot be used, such as boxing or mixed martial arts to assess training load. One limitation of Banisters TRIMP is that a standardised lactate curve is used in response to exercise which does not account for an individual athlete’s response to the training stimulus or mode [93]. This is catered for in Lucia’s TRIMP method by factoring in the individual athlete’s onset of blood lactate accumulation [94]. This, however, can create a monitoring method that is impractical for youth athletes due to the expense and experience needed for lactate testing.

Acute chronic workload ratio (ACWR) is the relationship between a negative function, fatigue, and a positive function, fitness, exploring what individuals have done and what they are prepared for [95]. This internal load measure helps to reduce the impact of some of the previously mentioned limitations of the RPE and TRIMP methods. Acute workload is the workload performed over a seven-day period which includes both training and gameplay [96]. This workload is commonly measured using session RPE (RPE multiplied by session duration) and given as an arbitrary unit which represents the fatigue aspect of ACWR. External load metrics such as total distance or number of sprints can also be used. Chronic workload, or the fitness aspect of ACWR, is the four-week average of the acute workload [96]. Following this, the ratio is calculated by dividing the acute workload by the chronic workload providing the ACWR [97]. This is classed as the rolling average model where absolute workloads are used, suggesting that each workload in an acute and chronic period are equal; although this fails to account for any possible decay in fitness or variations in the way in which load is accumulated [98]. The exponentially weighted moving average places a larger prominence on the most recent workload by allocating a decreasing weighting for each older workload value [99]. Unlike the rolling average, this model is designed to account for both the natural decay in fitness and the nonlinear relationship between injury occurrence and workload [100]. Once determined, the ratio gives an insight into an athlete’s preparedness [101]. If the acute workload is low, showcasing minimal fatigue, and the chronic workload is high, showcasing that the athlete has developed fitness, then the athlete is in a well-prepared state. In this case, the ACWR will be 1.00 or less [97]. If the reverse occurred, with high acute workload and low chronic training load, the athlete would be in a fatigued state and the ACWR would exceed 1.00.

Studies have investigated the use of the ACWR in a variety of sports such as rugby, AFL and soccer, with the majority concluding that there is a U-shaped relationship between ACWR and injury risk [96,101,102]. This means that an ACWR value under 0.80 shows a likelihood of undertraining and higher relative injury risk [97]. An ACWR value of 0.80–1.30 is classified as the ‘sweet spot’ in which workload has been optimised and there is the lowest relative risk [97]. Finally, an ACWR value greater than 1.50 is classified as the danger zone where the workload is high and there is the highest relative injury risk [97]. These figures are suggested guides and do not apply to every athlete and/or sport. Malone et al. [101] found the ‘sweet spot’ for the participating professional soccer players was between 1.00 and 1.25, and for rugby league players 0.85–1.35 [96]. Additionally, it needs to be noted that associations with injuries differ from predicting injury. Whilst associations such as relative risk can help illustrate the risk of a population, these statistical findings do not infer a prediction [103]. It has been shown that even strong associations may not predict if an injury occurs [104]. This was evident in a study conducted by Fanchini et al. [105] investigating the use of the acute chronic workload ratio for elite football players. The acute to chronic ratio, which was calculated for two, three and four weeks had a clear association with injuries (*p* < 0.05), however when analysing predictability, the receiving operating characteristics (ROC) curve revealed the area under the curve (AUC) was ≤0.60 [105]. AUC > 0.70 is reported to be needed to establish some prediction ability [106]. No such studies have been conducted amongst a female youth cohort. Dalen-Loresntsen et al. [107] explains that most ACWR studies report significant findings, but there are inconsistencies in the results and findings themselves. This may be due to variability in the methodology such as the recording of training load and the associated variables, how ACWR was calculated and the analysis of the relationship between ACWR and the associated variables [107].

Global positioning system (GPS) is a method of assessing training load and provides coaching staff with an innovative system to quantify overall distance travelled, speeds reached, both maximal and average, as well as distance accumulated at particular speeds [108]. GPS monitoring provides a comprehensive, valid and automated data collection method of an individual’s training load to ensure the required load is reached to elicit the desired adaptations whilst preventing overreaching [109]. This technology has been used considerably in many team sports such as rugby union, rugby sevens, Australian football league, hockey, soccer and cricket, each with its own individualised approach to obtain their desired data [110]. Currently, there is limited research on the use of GPS among young athletes. A study by Evans et al. [111] explored the use of GPS in a group of 26 female (13) and male (13) soccer players under 13 athletes and found that the average distance covered in a practice was 3.35 km, with a 56% increase in games. The discussion did not outline any specific differences between female and male participants. Moreover, few studies exist surrounding the use of GPS in a specific youth female population. Vescovi [112] explored the use of GPS monitoring in female youth soccer players and found that on average during a match they covered 6500–9000 m with positional differences being comparable to elite women. This study, however, did not discuss the validity of the use of GPS in young female athletes. A consideration that has been highlighted in the literature regarding the use of GPS monitoring in females is that if absolute thresholds are used to classify running velocity, these need to be based on female(s) athletes [113,114]. This is because male-specific thresholds could result in an underestimation of training load due to physiological gender differences in physical fitness and capacity [112,115].

The validity and reliability of GPS and its associated variables are impacted by the types of actions that are executed and the speed at which these actions are completed. The literature has illustrated that the margin of error typically rises when intensity and velocity rise, thereby diminishing validity [116]. Likewise, the reliability of these measures may also be decreased when utilised to monitor sports which complete a high number of directional changes [109]. Rampinini et al. [117] found that both validity and reliability increased to an acceptable level, (coefficient of variation (CV) = <5%), when monitoring overall distance covered and peak speeds for high-intensity intermittent exercise when GPS with higher sampling rates are used [117,118]. Coutts and Duffield [118] investigated the validity and reliability of six different GPS devices commonly used in team sports. It was concluded that when measuring total distance covered, all six showed an acceptable level of accuracy (CV = <5%) [118]. This result has again been supported by a 2019 study investigating the reliability of a 16 hz GPS within a rugby sevens team concluding that a CV of 0.5 ± 0.1 was present when assessing maximal sprinting speed [119].

The use of both objective and subjective measures is required to get a better representation of an athletes overall training load. When observing external load, individualised fatigue data from GPS analysis is considered a gold standard method of monitoring training, although it can be very expensive and time-consuming [110]. It must be stated that within an adult athlete population, GPS is useful to monitor aspects that affect these athletes the most, e.g., volume of high-speed running or contacts. For youth athletes, the aspects that pertain to high load markers that are measured through GPS are either minimised or regulated at a youth sport level, e.g., ripper rugby vs. tackle, decrease pitch sizes or playing times. Therefore, at a young athlete level where these adaptations are put in place, GPS may only be useful in any return-to-play monitoring or if external load monitoring is important to monitor progression. Additionally, no focused research has been conducted regarding the reliability and validity of GPS in a youth population, and more specifically female youth. Conversely, sRPE is non-invasive and cost-effective, which provides an advantage for a youth population as sport is not as highly funded, making it a practical choice for youth athletes. sRPE has also been shown to be a valid measure of tracking internal training load within youth athletes [84]. However, when excessively used, difficulty can arise when determining worthwhile changes in sRPE, thereby highlighting the possible usefulness of ACWR to provide an easy understanding of collected data which is needed in a female youth population.

### 2.5. Recovery Monitoring

Athletes have differing recovery rates and therefore will have different tolerances to both training stress and other life stressors. Monitoring recovery can provide objective and subjective data, depending on the method used, which can quantify the stress an athlete is under and whether they are recovering optimally. Recovery is a multidimensional process which is complicated further when working with youth athletes as a result of multiple sporting commitments as well as a high potential for stressors to occur [2]. Physiological stress and the subsequent recovery of an athlete are largely controlled by the autonomic nervous system and therefore, common recovery monitoring protocol involves observing and analysing an individual’s heart rate or elements thereof [120]. Heart rate variability (HRV) monitors the changes in cardiac autonomic activity which is deduced from vagally mediated HRV [120]. This monitoring practice has been shown to provide an insight as to how athletes are responding physiologically to training, regardless of their subjective perception of recovery [120,121]. HRV is measured over a week, through either daily fluctuations (analysed through the coefficient of variation), or through average cardiac vagal activity, and has been stated to be sensitive enough to indicate running performance and overall response to training load [120].

A ten-week study on elite triathletes found that the trends of both daily fluctuation and absolute HRV provided beneficial information which was able to suggest progression towards maladaptation and non-functional overreaching [122]. Comparable results have been reported as a three-week study conducted in a women’s soccer team investigating HRV and training load found that both the mean and the coefficient of variation of an HRV measure was a sensitive marker to changes in training load [123]. Studies have shown that individuals who have demonstrated fatigue and subsequent poor responses to the prescribed training load also had reductions and larger day-to-day variations in vagally mediated HRV [120]. The reliability of HRV has been explored by Nakamura et al. [124], reporting both the intraday and interday intraclass correlation coefficient as 0.96 and 0.90, respectively, therefore demonstrating a high level of reliability. HRV has also been reported to be a valid measure (r = 0.92) when using a smartphone application; however, both reliability and validity are dependent on the protocol and analysis methods used, supine vs. standing measurement or smartphone vs. computer software [125].

Dissimilarly to HRV, questionnaires are often used to capture athletes’ perspectives on recovery and readiness to perform. A commonly used questionnaire to assess recovery levels in athletes is the Recovery-Stress Questionnaire for Athletes (RESTQ-sport) [126]. This questionnaire consists of both general and sport-specific stress and recovery scales, with a focus on the frequency of stress and recovery behaviours as well as psychophysical states [127]. RESTQ-sport has predominantly been used for endurance athletes such as rowers, cyclists and triathletes [128,129,130]. Coutts et al. [131] reported that in a group of 16 triathletes who completed four weeks of overload training, the RESTQ-sport illustrated an impaired recovery-stress state as training load increased, followed by an improved recovery-stress state during a taper. Along with being used in several sports in adult athletes, the RESTQ-sport has successfully been used to monitor recovery and stress regarding training load in youth athletes. Kellmann et al. [132] assessed the stress and recovery of female and male junior rowers during the preparation for the World Championships, the RESTQ-sport was able to quantify the effect training load had on the athletes’ recovery and stress. RESTQ-sport has also been reported as a valid measure and to have acceptable reliability; however, limited studies have been conducted surrounding both the reliability and validity, suggesting more research needs to be conducted to draw this conclusion [127,133].

Another common subjective recovery monitoring tool is a visual analogue scale (VAS) specifically used to assess pain and muscular soreness. VAS requires an individual to mark a point along an unmarked 10 cm line which they believe represents how they feel about different questions [134]. Most commonly, VAS is used to assess and quantify musculoskeletal pain and has been used in both sporting and medical settings [135]. Within a sporting and exercise setting, VAS is typically used when investigating delayed onset muscle soreness as an individual can report their subjective level of muscle soreness [134,135,136,137]. Furthermore, VAS has also been used in patients recovering from surgery, where it was reported that a statistically significant difference score in VAS equates to a clinically significant change in pain [138]. VAS has been shown to have high reliability (ICC = 0.99) and validity (r = 0.84–0.97) [139,140]; however, VAS has not been explored as a tool for monitoring recovery and muscle soreness within team sports and is yet to be used in (female) youth. It has, however, been used to report pain levels in males and females and showcased that females will report more negative pain experiences than males [141]. This highlights a consideration that needs to be acknowledged if using VAS, as males and females have different perceptions of different sensations, validation data that were acquired through male participants may not accurately represent female participants.

The practical application of recovery monitoring needs to be considered when monitoring (female) youth athletes. Although HRV provides beneficial information about an individual’s recovery, it requires a device to measure and record heart rate as well as an understanding of meaningful changes within the data. The RESTQ-sport is a simpler tool but due to its length, may be more suitable for weekly application and therefore may fail to identify acute changes [142]. Finally, much like the RESTQ-sport, VAS is a simple and practical approach to monitoring recovery; however, to date it has only been used to assess pain, thus creating a need for VAS’s ability to assess multiple aspects of recovery to be explored. Although the current time and financial implications of the mentioned monitoring strategies may dampen the practicality for some, recovery information is vital and therefore a method should be chosen and adapted to best fit the group or individual.

### 2.6. Injury Reporting

Sport-related injuries are on the rise with increased sports participation, with sport being the leading cause of injuries among youth in several countries [143,144]. In 2017, New Zealand’s Accident Claim Corporation (ACC) reported over 90,000 sport-related claims being filed for youth 19 years old and under: a 60% increase from 2008 [145]. Injuries sustained through sport are now being identified as a barrier for youth to continue playing sports. A 2015 systematic review of dropouts in youth sports found that injuries were the second most reported structural constraint causing youth to drop out of sports, second to time constraints, possibly due to a lack of self-efficacy or fear of reinjuring when returning [8,146].

Sporting injuries can be split into two main groups: acute trauma and overuse injuries [144]. Acute trauma is the result of a specific impact or event, typically to one area of the body, whereas overuse injuries are defined as cumulative injuries with damage caused by repetitive submaximal demand without adequate rest over time [144]. Overuse injuries in youth sports are particularly prevalent as a result of many young athletes playing more than one sport. It is estimated that overuse injuries comprise 45.9% to 54% of all injuries sustained through sport, with the prevalence in specific sports ranging from 37% for skiing to 68% for running [7]. Leppanen et al. [147] investigated the prevalence of overuse injuries in youth team sports and found that out of 387 participants, 204 overuse injuries were registered equating to 1.51 injuries per 1000 h of exposure. The most common injury sites are the knee (35%) and the lower back (21%) with 44% of injuries being classified as severe [147]. Moreover, the risk of certain injuries is also heightened for females due to structural and hormonal differences [148].

Frisch et al. [149] investigated sex-specific injury patterns and risk factors in young high-level athletes and found females had a higher proportion of injuries to the ankle and/or foot in comparison to males (34.8% vs. 16.8%). This is a result of females having greater joint laxity, and poorer proprioception and coordination in comparison to males, consequently increasing their risk of ankle and knee injuries [148,150]. Females represent a high proportion of anterior cruciate ligament (ACL) injuries, with 37% of all knee injuries being ACL related, in comparison to 24% for males. Various factors have been suggested to cause this increased risk of ACL injuries in females, such as increased joint laxity, as well as wider pelvises producing a greater Q angle which may be a predisposing factor for knee weakness and instability [143,144,151]. In long-term studies on youth athletes who underwent meniscus surgery, over 50% will have knee osteoarthritis as well as pain and functional impairment [152]. Consequently, these injuries not only lead to youth withdrawing from sport; the long-term effects of child and adolescent injuries are largely unknown. As a result of this, injury monitoring is becoming more prevalent in sports as injuries are not always caused by a single event and the long-term effects of these injuries are often unknown. However, injuries cannot be monitored the same as variables such as training load as a singular injury does not need to be further quantified, whereas a training session can be broken down and quantified in several different metrics.

Injury reporting is a broad term that encompasses surveys, registers and surveillance, each having its own benefits. Surveys are completed either as a ‘one-off’ or at set intervals to collect detailed data but are at risk of recall bias as they rely upon a participant’s honesty and memory [153]. Registries are the collection of data regarding a particular injury or case in a set population, thereby preventing the ability to specify and tailor prevention plans to an individual team or athlete [154]. Typically, the literature focuses on injury reporting or surveillance, which is the ongoing collection of data regarding the athlete, i.e., what the injury was, when it happened and how it happened. One aspect of injury surveillance is the classification of injury severity. This is classified according to the playing time lost, e.g., transient meaning no training missed, minor is up to seven days lost, moderate is eight to 28 days missed, or major which is ≥29 days lost [155]. Injury surveillance also allows for injury occurrence to be calculated by dividing the total number of injuries by total injury exposure and then expressed as rates per 1000 training hours [155]. It is noted that the application of injury surveillance and the information gained is pointless if it is not further analysed to determine trends or tied to prevention interventions [153]. As a result, the purpose of this data collection is not based on direct performance enhancement, it needs to monitor any trends that arise in injuries, to further evaluate potential causes for injury [153,154]. These injuries can further be analysed against other monitoring variables, such as training load to identify any influence this has [153,154].

**Figure 2:**
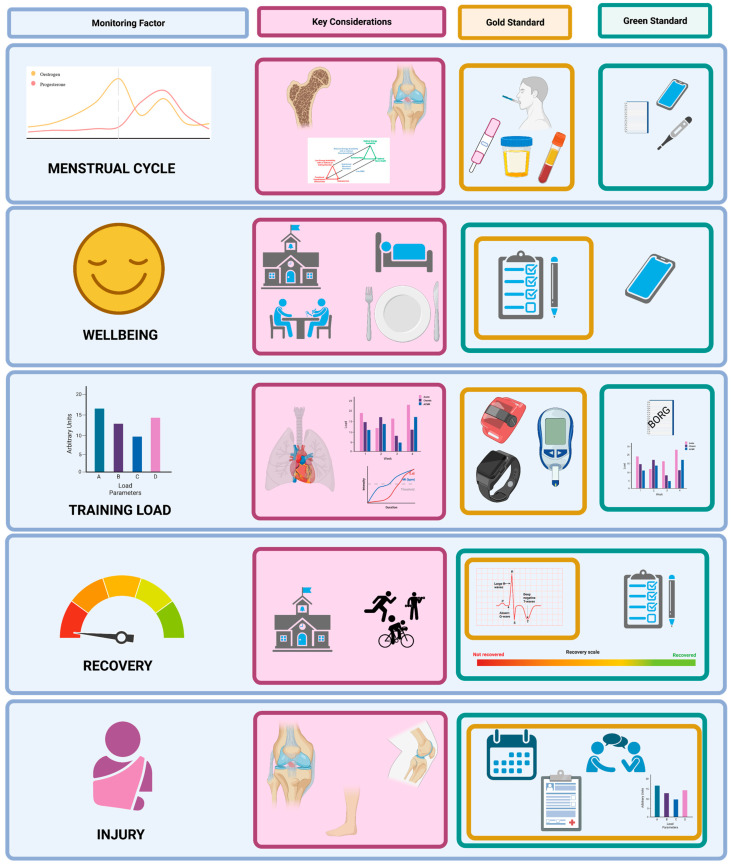
Monitoring factors, their respective key considerations in youth female athletic populations and gold and green standard monitoring practices for each factor. Note that some tools can be used to monitor multiple factors simultaneously, or will be used at differing monitoring intervals to provide contextual depth of understanding. Gold and green standards may overlap when low-cost options are reliable and valid measures of the monitoring factor under consideration.

For a monitoring system to be successful and applicable, the information that is reported must be valid and reliable. This is often an area of weakness for injury surveillance as Whatman et al. [156] reported that of the youth athletes surveyed, 87% admitted to either downplaying or hiding an injury during a game, of these athletes, 26% disclosed that they did this often. Importantly, a 2005 study investigating the validity of the injury surveillance system found that when the designated team physiotherapist or sports medic completed an injury report form there was a 96.2% completion rate, in comparison to the physicians’ diagnosis form which had a 36.4% completion rate [157]. This was explored, and has only been explored, in a mixed sex cohort where possible difference in the accuracy of female vs. male reporting was not identified. Simply, for a successful and practical approach to injury surveillance, it is recommended that reporting and recording is completed within the team, and strong team communication is emphasised to ensure all injuries are accounted for (Figure 2).

## 3. Conclusions

It is paramount that specific conclusions, practical applications and gaps in research for the monitoring of youth female athletes are highlighted as the preponderance of research surrounding training load monitoring has been conducted on male adult athletes, which cannot be directly applicable for female youth athletes.

For female youth athletes, a reliable and accessible solution to tracking and monitoring menstrual cycles needs to be used. In this population, the combination of a counting back method and education surrounding symptom tracking around phases of their cycle is recommended through wellness questionnaires. This may be able to provide both a retrospective and prospective insight into the individual impacts of their menstrual cycle on their training performance and recovery. Another practical application surrounding menstrual cycle monitoring is to invest extra effort into informing and educating parents/guardians of female youth athletes about the energy/nutritional requirements of their age and sports to reduce the risk of RED-s and the subsequent impact this can have on reproductive health and bone density.

It is important to ensure the monitoring protocol has strong practical applicability and is creating an individualised approach. As previously stated, a large quantity of training load studies is completed on a male cohort and therefore produce male-specific data [19]. This can lead to an over or underestimate of training load as physiological and loading differences exist between genders [112,115]. Subjective measures already allow for an individualised approach as players assess their own exertion, recovery and wellbeing against their own internalised standards, whereas objective measures may not be as readily individualised. For this reason, objective measures that either have valid and reliable female data sets and/or can be individualised need to be used. For example, GPS can be designed to allow for a more individualised approach, e.g., an athlete’s maximal speed can be used to create specific speed zones for their GPS monitoring, creating relative data as opposed to absolute data [158].

Further quality research on the menstrual cycle impacts on performance and injury is needed. Within this research, attention should be given to the potential differences in the impact (or lack thereof) of their menstrual cycle in relation to how long the athlete has been experiencing their cycle, e.g., whether a recent onset of menarche has more or less of an impact on performance and injury in comparison to a menstrual cycle that has been experienced for several years. Supplementary research around a more targeted insight into LTAD from a female perspective relating to the coaching considerations and challenges should be investigated. It is known that females enter the LTAD stages earlier than their male counterparts. However, a more targeted insight into sex bias adaptions secondary to male vs. female age of transition from each stage may support practitioners to be more dynamic in their facilitation of LTAD and more specific to the important variable to monitor for a female youth athlete [159].

From a more generalised viewpoint, there is no single marker that can provide global information regarding an athlete’s recovery and wellbeing; much like there is no one test when used in isolation, that can provide a well-rounded picture of an athlete’s abilities [160]. For this reason, it is advised that a comprehensive monitoring protocol should capture both internal and external load, employing both objective and subjective measures [3]. In addition to a comprehensive monitoring protocol, attention needs to be given to methods of improving adherence, as without strong adherence data can neither be reliable nor valid, potentially impairing effective adaptations to load and recovery [13].

Within a youth athlete population, adherence can easily be impacted by a lack of money to continuously provide expensive equipment, inexperience in understanding data, and poor “buy-in” from coaches and/or parents [3]. Therefore, for youth athletes, in particular those not at an elite level, the monitoring protocol needs to be simple and inexpensive whilst providing beneficial information. Hence, sRPE, the RESTQ-sport and customised questionnaires are deemed practical options for this cohort [18]. More specifically, questionnaires should fit the time requirements and needs of the team and may be best conducted once to twice a week as opposed to daily, further ensuring the data generated are more manageable [70].

Following data collection, coaches and support staff need to have an established method of determining meaningful change or “red flags” in the acquired data to determine whether there will be a significant negative or positive impact on performance and health [3,70]. This threshold will differ between each method and potentially each individual; therefore, in youth sports, it is again vital for data to be easily understood and presented, especially as these groups may not have dedicated sports scientists or practitioners. Consequently, in youth sports, there is a lot of room for future research to be conducted, to establish whether the smallest worthwhile change is the same as within an adult population or is specific to youth athletes. Knowing and understanding how to interpret worthwhile change is essential in a monitoring system to provide meaning and a practical purpose to data collection.

The validity and reliability of subjective measures in youth athletes also requires future research. This cohort may be less capable to accurately judge their perceived recovery, exertion and wellbeing as a result of less experience doing so and less comprehension of their limits [1]. Validity studies have been completed on the use of RPE in youth, determining that it is a valid measure; however, minimal validation studies looking at subjective recovery and wellbeing studies have been carried out in a youth setting [86,161,162]. It has been illustrated that there may be correlations between training load and wellbeing, which provides information surrounding players’ recovery and mental state. However, few studies comment on a possible correlation between performance measures (power output, jump height) and well-being, and whether a decrease in well-being decreases these performance measures [72]. Conversely, this could be explored regarding whether an individual or team’s success affects a player’s subjective wellbeing. This is especially relevant in youth athletes as these athletes experience additional stress when they believe they have performed poorly [63].

A similar avenue for future research is the role of academic load in performance, both in terms of performance measures and a team and/or individual’s success. During times of high academic load, athletes are more inclined to become ill or injured; however, the influence of academic load on how successful, subjectively and objectively, an athlete is in their particular sport(s) is yet to be documented [63]. In addition to the complexity of training and monitoring youth athletes due to the stress and load of schooling, these athletes typically compete in other sports, participate in physical education and may perform their own training outside of their sports [2]. For this reason, capturing the proportion of training load that is obtained from one sport, in comparison to athletes’ other activities, is recommended. This may provide useful information regarding the extent to which a coach needs to consider athletes’ other training habits, to optimise their training and recovery.

## Data Availability

Not applicable.
